# Mental health and well-being in older women in China: implications from the Andersen model

**DOI:** 10.1186/s12877-020-01639-z

**Published:** 2020-07-25

**Authors:** Hui Yang, Aaron Hagedorn, He Zhu, Honglin Chen

**Affiliations:** 1 Department of Sociology, Minzu University of China, Beijing, China; 2 University of Texas at Arlington School of Social Work, Arlington, TX 76019 USA; 3 Faculty of Humanities and Social Sciences, Bejing University of Technology, Beijing, China; 4 Department of Social Work, School of Social Development and Public Policy, Fudan University, 220 Handan Road, Shanghai, 200433 China

**Keywords:** Mental health, Andersen model, Older women, China

## Abstract

**Background:**

Mental health and well-being among older women is an important topic due to the feminization of later life as women tend to have longer life expectancy resulting in elderly women being more advanced in age and outnumbering men. Older women generally play a key role in their families lifelong and mostly depend on social support from their family and close friends in older age to cope with any limitations they face as a result of age-related changes in their health and functional ability.

**Methods:**

We examine which factors predict mental health and well-being in older women using the Third Wave of the 2010 Female Social Status Survey conducted by the All-China Women’s Federation (*n* = 3527). Applying the Andersen Model, regression analysis exploring predisposing, enabling and health need variables were tested using SPSS version 22 predicting a mental health scale.

**Results:**

Results showed that living with a spouse was not a significant predictor of mental health for women, while it was for men (b = − 1.2, *p* < .01), ownership of property is significant only for men (b = −.96, *p* < .05), whereas women’s mental health is more strongly predicted by current exercise (b = −.89, *p* < .01) and participation in leisure activities (b = −.69, *p* < .001). Close relationships with neighbors, qualifying for old-age benefit programs and being in better overall health supports positive mental health for both men and women. Reporting delayed medical treatment is associated with a negative impact on mental health for men, but oddly women who report the same actually report better mental health, perhaps suggesting older women take pride in their self-sacrifice.

**Conclusion:**

The findings of this study suggest that gender differences in wealth, living alone, and social participation are interpreted differently by women, who have longer lives with generally fewer material resources. Enabling factors tend to be more associated with financial factors for men, while women rely on a social convoy to thrive longer than their male counterparts.

## Background

People of different social classes, ages and cultures may have very different expectations of the human and financial resources needed to sustain their retirement years. Losses of continuity as age-related physiological and psychological changes take shape in older age may *predispose* some older adults to more rapid decline [1]. Access to family, supportive social services or neighbors can create an *enabling* social convoy (Antonucci, Ajrouch & Birditt, 2013) of accessible caregivers and sources of social support. With age and cumulative disadvantage [2] *need* factors which affect the amount of support required can diverge over time across individuals as physical and/or cognitive aging progress.

The declining expectations of social experiences at advanced ages described by socioemotional selectivity theory predicts a narrow but close social group can provide comfort to older adults [3]. In addition, among Asian older adults, there may be a special concern about being a burden to others creating an anxiety and source of concern [4]. China offers a unique perspective on mental health as its culture has long held fast to traditions and obligations which create very strong social norms. With little history of retirement planning, older people rely on a sense of a safety-net support from their adult children, though as cost of living increases, many older adults find they invest significantly in their children and grandchildren, with uncertain returns on that investment in the future. Given the clear filial obligations, laws which make adult children financially responsible for their elder parents, rapid inflation, and the relatively low incomes of most older adults (with mandatory retirement for many between ages 55–65), older adults may feel they become a burden on others. The older generation has far fewer years of formal education, and the generation gap in lifestyle and life quality expectations may be quite different across the age range.

In this paper we choose to focus on measuring mental health and sense of well-being among older adults in China who have lived through many dramatic changes in society including the Cultural Revolution during their childhood, rapid industrialization during their adult lives, and rapid inflation in later life [5]. The one child policy since 1978 dramatically reduced family size and social support available to the older generation. Confucian filial piety values have declined as a result of improved living standards and increasing financial prosperity for today’s middle generation. However, there is now a rising percentage of older adults with unmet social support needs as relatives live further apart [6]. While China’s development has benefitted urban dwelling working age individuals, older adults are at the mercy of their children and close relatives who provide most of the cash and resources necessary to function. The result is 40% of elders in a national sample of those 60 and older in China reported symptoms of depression [7], compared to estimates in the US of only 5–20% (Blazer et al., 2003) reporting significant depressive symptoms for at least 6 weeks.

The concept of mental health is unique in China, as the cultural revolution (1966–1976) nearly erased psychological diagnoses, which were seen as incorrect political thinking [8]. The social environment of China was completely different when today’s 60 and older generation were socialized as children. The impact of the childhood environment can have profound impacts on mental and physical health (Brandt et al., 2012). To this day many prefer to avoid the stigma of depression, despite the fact dramatic shifts in society have left many in relative poverty. The residual effect of their challenging life can be seen in some surprising empirical results. For example, living alone appears to be associated with depression for older women but not for older men in another large study in China [9]. Some of the traditional risk factors for depression may operate differently in China due to the high level of resilience often found in those who have survived extraordinary adversity [10–13].

Understanding mental health requires an in-depth analysis of the environment and personal characteristics. The Andersen model was originally proposed as a model to predict health care utilization (Fan, 2014). However focusing attention separately on predisposing, enabling, and need factors makes sense when investigating multi-causal pathways influencing one’s locus of control and perceptions of the world, with the ultimate impact on behaviors and actions can be used as a general model of access to support in the community including social support and caregiver support [14]. Furthermore, perceptions of mental health can affect actual health [15] and may predict service utilization [16] as well as social or environmental support need (Zheng, 2015). Therefore, the grouping of predisposing, enabling and need characteristics is a useful conceptualization of the potential triggers of poorer reported mental health, and the need for a support system should needs arise. Further, this model could be used to estimate the resources needed to manage those with higher level support needs.

Older women are at greater risk of physical disability and sleep disorder than their male counterparts (Barreto, Giatti, & Kalache, 2004 [17];; Daglar et al., 2014), which may affect their ability to manage later life stress and anxiety (Arber & Ginn, 1994). Poverty is also feminized as the income gap between older women and older men grows more extensive over time (Pearce, 1978 [18];). Nearly all older women across China are dependent on family support due to limited income to support for their retirement years. Declines in family size and migration strains that family dependence. Older women depend on their family members to provide income and care resources (Freid, Prager, MacKay & Xia, 2003 [19];).

### Life course cumulative disadvantage among elderly women caused by gender difference

Older adults represent the impact of life-course cumulative disadvantage in elderly women’s lives [20], and cumulative disadvantage (Crystal & Shea, 1990). Despite recent social welfare policies that protect older people, it should be noted there remains a pension gap between elderly men and elderly women as well as other gender difference issues that have recently been highlighted by researchers [17, 21, 22]. The major gender difference in social welfare benefit is the fact that older men are still the predominant beneficiary of social welfare policy, while older women are relatively protected at a low level with fewer benefits (Jia, 2006). The percentage of older men living below the poverty line (RMB 261.65 per month) is 9.7% in urban areas while that rises to 43.1% in rural places [23]. However, older women in urban areas are 3 times more likely to be in poverty than older men as 41.1% of older women live in extreme poverty in urban areas, while 65.5% of rural women live on less than 93.9 RMB per month [23]. Therefore, compared to older men, older women are more likely to fall into poverty, unable to sustain their former life situation with impaired social functions, and have a lower standard of living.

When exploring what causes the relatively vulnerable living status of elderly women, social factors can be just as important as individual factors in the discussion framework. For example, some researchers believe that since the current pension system is directly attached to employment status, in which the gender perspective plays a role in determining pay, therefore pension systems exacerbate the poverty problem among elderly women [24, 25]. Most women will be widowed in late life because they are generally younger than their husbands and are expected to live longer lives (Bennett, 1997), leading to many living alone and with very low income (Xu, 2001). Thus, lack of care resources among older women can make a great difference in their later life. Furthermore, elderly women still need to bear the responsibility of attending to the family, especially taking care of their grandchildren, which not only makes it less possible for the elderly women to receive care from family, but in turn adds extra burdens to them (Eaton, 2005). In summary, multiple factors can be used to understand the unique challenges women face later in life as a result of low financial resources, limited family support, and a lifetime of cumulative disadvantage.

### Conceptual framework, Research Question & Hypotheses

Employing the framework from the Andersen’s behavioral model of health services use in the context of population level health outcomes across China, we explore how an older adults’ mental health and sense of well-being is a function of three characteristics, namely: predisposing, enabling, and need factors. Predisposing factors in the current study include individual demographic characteristics that influence older age support needs and an assessment of participants’ recognition of mental health. Enabling factors refer to personal and societal resources that may facilitate positive mental health support. Need factors pertain to the assessment of one’s condition and are generally objective, or involve professional evaluation of needs, or identified diseases or relevant caring demands.

**Fig. 1:**
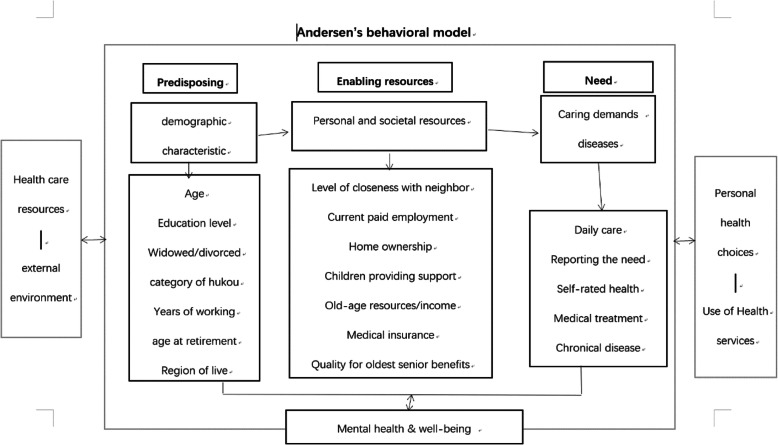
Conceptual framework of Andersen’s Model on mental health and well-being

In this study, we examine which factors predict mental health and sense of well-being in older women. Our research question is how do reported mental health scores differ by predisposing factors (i.e., issues outside of one’s direct control that define the opportunities available to either enhance or decrease quality of life), enabling factors which demonstrate an ability to navigate through society (e.g., having a job, owning a home, available social support, access to social and medical resources) and need factors (which demonstrate actual life challenges which could lead to a need for support or lead to anxiety about one’s well-being). We are particularly interested in how mental health and sense of well-being differs by gender between three steps. First, this paper examines the effects of the characteristics that affect one’s identity in determining their sense of well-being. Next it examines how one’s identity in society is enacted through societal roles and how that influences perception of mental health. Finally, we examine how those coping with actual life problems report their mental health score. The following Fig. 1 illustrates the research framework of the current study on the basis of Andersen’s model.

### Hypotheses

The first hypothesis is those living in the most resource poor environments have the lowest mental health scores. The second hypothesis is those who retired at the youngest ages have the lowest mental health scores. The third hypothesis is enabling factors should differ greatly between men and women as reflected in their life course experiences with different expectations and roles for men, with older women likely having weaker associations between socioeconomic measures and their mental health scores. Thus, we expect that home ownership and being currently employed past the retirement age is negatively associated with mental health scores for men, while women may have no association between mental health scores and socioeconomic factors.

Need factors include the presence of chronic diseases or physical limitations that could add strain on quality of life. Aging is often associated with needing supportive assistance. Thus, the fourth hypothesis is having daily care needs or needing to delay medical treatment are also strong predictors of mental health scores.

The equation which describes this regression analysis is:

$$ {y}_i=a+b^{\prime }{Z}_i+{c}^{\prime }{v}_i+{d}^{\prime }{x}_i+{u}_i $$

Where, *y*_*i*_ is the outcome of self-rated health, where *i* indicates the individual, Z is the predisposing factors . *v* is a vector of variables represented as Enabling factors, *x* is a vector of variables represented as Need factors, and *u* is the error term.

## Methods

### Sample description

The data were collected during the Third Wave of the 2010 Female Social Status Survey conducted by the All-China Women’s Federation and the National Bureau of Statistics [17, 26]. The sample which included 3527 older people ages 65–98 years old include detailed questions about nine aspects of health, education, economy, social security, politics, marriage and family, lifestyle, legal protection and awareness, and gender awareness and attitudes. Individual surveys were developed for children, senior citizens, college students, people with migration experience and high-level talents. The original survey collected 105,573 questionnaires filled out by respondents aged 18 and over. A subset of 3527 individuals who were 65 or older and represented all Provinces of China answered an additional questionnaire focused on the unique issues of aging.

Other researchers using this data have reported mental health scores among men to be better than their female counterpart across all 31 provinces and major autonomous cities in China (Wu, 2011). Those in an empty nest had lower mental health than those living in multigenerational housing ([7]; Wu, et al., 2015). We build on those findings and further explore how proximity to supportive community and family resources influence mental health across the various regions of China.

### Measures & statistical methods

In this study, mental health and well-being was measured as the sum of mental health factors asked in the survey which were measured on a 5 point Likert scale including the following questions: “I can deal with my emotion no matter what happened” “I always feel nervous and afraid” “I feel lonely very often” “I make my own decisions” “I feel useless” “I love to socialize” “I am willing to and able to help others,” and “I am willing to learn new knowledge”. The scale was coded with higher scores reflecting negative thoughts. While the individual factors capture ideas ranging from anxiety to interest in socializing, engaging with others, and seeking out new resources and information, the combined scale has high reliability with an Alpha value of 0.76 suggesting these factors share a similar central construct. The questions are highly similar to what is found in the Geriatric Mental State Exam, which has been used in China before (Chen et al., 2002; Copeland et al., 2004). This measure has been tested in the Chinese population in several studies using s that measure issues of anxiety and depression [27, 28], as well as locus of control [27–29].

The variables representing *predisposing* factors are age, education level, widowed/divorced, category of hukou, years of working, age at retirement, region where you live and ability to manage your income. The variables representing *enabling* factors are: level of closeness in relationship with neighbor, current paid employment, whether one owns a home, children providing social or financial support, and qualify for old age resources, have medical insurance, qualify for oldest senior benefits. The variables representing *need* factors are: reporting the need for support in daily care, self-rated health, whether you’ve delayed medical treatment, and the number of chronic diseases. Each of these above factors are found in previous model of literature [30].

IBM SPSS version 22 was used to perform linear regression to predict the mental health score. In order to do this the mental health needs variable was recoded as a scale variable by summing the responses to the 8 negative mental health events depicted above. As each response ranged between 1 (good) to 4 (poor), the summed score measure ranged from 8 to 28 (m = 15.27, SD = 3.85). Multilinear regression was performed using predisposing, enabling, and needs factors as predictors of mental health and well-being, run separately by gender.

## Results

### Demographic characteristics

The sample is representative of the older population in China, and thus 12.2% of respondents are from major cities including Beijing, Tianjin and Shanghai, while 30.7% are living in the 8 Eastern Provinces, 30.3% from the central 8 provinces, and 26.8% in the 12 western provinces which surround the major cities but do not include those in the cities. Approximately 47.6% of all the elderly respondents hold an urban Hukou. The majority (74.3%) of the older people were born in a rural area, with only 13.5% of the respondents born in a town or county, and another 12.2% in a city. Non-Han respondents only represent 7.8% of the sample, as would be expected. Approximately 68.9% of respondents were educated at or below an elementary school level, 25.7% were educated at junior high or high school/secondary technical school level, 3.1% at junior college, and only 2.2% hold a college or higher degree. Most respondents are either married and living with their spouse (61.6%) or widowed (33.7%), only 2.4% are married but living apart from their spouse, while 1.1% remain single and the remaining 9% are divorced. The demographic information of the sample is shown in Table 1.

**Table 1:** Demographic information of the sample (*N* = 3527)

Item	Frequency	Percentage (%)
Sex		
Male	1809	51.3
Female	1718	48.7
Hukou		
Urban	1679	47.6
Rural	1846	52.3
Missing	2	0.1
Education Level		
Low Literacy	1296	36.7
Elementary School	1135	32.2
Junior High School	595	16.9
High School/Secondary Technical School	309	8.8
Junior College	109	3.1
College and above	78	2.2
Income		
Females	7920.14 Yuan, SD = 14,962.23	
Males	12,708.08 Yuan SD = 14,977.20).	

### Distribution of mental health and well-being by gender and region

Given the vast range of lifestyles across China and the different availability of social support from family or community services, firstly, we explored whether region plays a significant and perhaps different role for men and women in predicting mental health. Mental health scores vary by region, with those living in the coastal cities more likely to report good mental health, and those living in the central and western provinces disproportionately reporting worse mental health. The more resource rich environment of the larger cities has a generally positive effect on the reported mental health of older adults (Table 2).

**Table 2:** Mean Value of Mental Health Scores by Region

	Large City	Eastern Provinces	Middle Provinces	Western Provinces
	*M SD*	*M SD*	*M SD*	*M SD*
Women	14.1 (3.6)	15.4 (3.9)	16.1 (4.1)	16.1 (3.8)
Men	13.6 (3.3)	14.6 (3.6)	15.5 (3.7)	15.4 (3.9)
*F (df1, df2) p* *N*	2.09 (1, 409) .150 411	11.11 (1, 953) .001 955	6.54 (1, 938) .011 940	7.38 (1, 800) .007 802

As shown in Table 3, nearly everyone had a child, and the majority of the sample reported they had the financial and often daily care support of at least one child. While most had few children, with 53.2% reporting between 1 and 3 children, a large proportion, 42.1%, reported between 4 and 6 children, and 3.8% with 7 or more, and only 1.3% reporting 0 children.

**Table 3:** Number of children by gender

Gender	no child	1–3 children	4–6 children	More than 7 children	Total
Men	1.1%	57%	38.8%	3.4%	1775
Women	1.5%	48.5%	45.4%	4.6%	1716

The first hypothesis proposed those living in the most resource poor environments (Western provinces) would have the lowest mental health scores, and while this was supported for men, it was less strongly associated for women. Furthermore, men benefitted from support from a child, whereas women generally did not show an association between availability of support and their mental health scores. Respondents living in lower income middle and western provinces reported mental health scores that were 2 points higher (worse) on average than found in the large cities (*p* < .01). While women generally scored 1 point higher on the mental needs scale than men in the same region, the difference by gender is not statistically significant however. While middle and western provinces tend to have larger family sizes, young people tend to move further away in search of work, and have lower incomes to return to their families and thus those in the middle and western provinces tend to report higher concerns even after controlling for support from children (Table 4).

**Table 4:** Regression of region on mental health score controlling for availability of support (Eastern provinces is reference)

	Male	Female
intercept	2.62	2.542
Large city	−.372*	−.375*
Middle province	.201	.195
Western Provinces	.212*	.175
Support from Child	−.242*	.032

*Indicates to significant factors, *p* < .05 or < .01 level

### Predisposing, enabling, and needs factors explaining the pattern of mental health and well-being

#### Predisposing factors

The regression results in Table 5 show having a rural hukou has a very powerful effect on social stress and a negative factor affecting mental health for older adults, with rural hukou men reporting scores more than 1 point higher than their urban counterparts, and women nearly a point higher even after controlling for other demographic characteristics. Completing another level of schooling on the education scale has a protective of nearly 1 point (female b = −.702, male b = −.778) on the mental health scale. Thus, those who are both rural hukou and in the lowest education category generally report worse mental health scores. Region was explored to test it impact while controlling for the other factors, and only the middle provinces standout as substantially worse (female b = .642, male b = 586).

The second hypothesis proposed those who retired at the youngest ages would have the lowest mental health scores, and this was not supported. On the issue of time since retirement (male b = .405) and number years working, only men who were more recently retired reported better scores on the mental health scale (no effect for women) and men who worked a greater number of years also reported better mental health scores (male b = −.682). Prior work might endow an individual with more savings and a sense of purpose; though the meaning of work may be very different between men and women as many older women devoted most of their working-age years to child care. We also investigated one aspect of cognition and empowerment, the ability to manage one’s finances. The effect was the same for men and women, lowering the scores on the mental health scale by nearly 1 point (female b = −.735, male b = −.751).

Widowhood disproportionately affects women’s mental health, with widowed women scoring 1 point higher while widowed men score only half a point higher than their non-widowed counterparts. It should be noted also that women were far more likely to be widowed, 48.3%, compared to 19.8% of older men Women were more likely to report needing assistance (28.6% vs 26% of men, *p* < .05) and more likely to report a lack of assistance when needed (7.6% vs 6.1%, of the older male population, *p* < .05) (Table 5).

**Table 5:** Regression of Mental Health Score on Predisposing Factors

	Female	Male
Constant	15.92	15.64
Age (in 10 years)	.508^a^	.617^a^
Education level	−.702^a^	−.778^a^
Widowhood	.891^a^	.528^a^
Hukou	.**632**^**a**^	1.15^a^
Retire age	−.266	.405^a^
Number of Years worked	−.133	−.682^a^
Manage income	−.735^a^	−.751^a^
Big cities	.068	−.136
Middle province	.642^a^	.586^a^
West	.277	.417

Note: ^a^ Indicates to significant factors, *p* < .05 or < .01 level

#### Enabling

The third hypothesis, which proposed enabling factors should differ greatly between men and women with older women likely having weaker associations between socioeconomic measures and their mental health scores was only partially supported. As shown in Table 6, the meaning of paid work may vary by gender. Paid work in old age may be a sign of poor financial condition in old age, as it is associated with lower mental and well-being (b = .65,8 *p* < .05), though it should be noted only 13.3% of women reported current paid work while no effect is seen for the 24% of older men who are currently working (*p* = .227). An interesting difference between women and men is found in home ownership with a nearly 1-point lower score on the mental health questionnaire for older men who owned a home. There is no impact of home ownership on older women despite the fact 53.3% of women reported owning a home. Though men were indeed more likely to report owning a home, with 76.8% reporting ownership, the impact of this home ownership varies. While there is some variation in home ownership rates across the regions (49.4% of women in Eastern provinces to 58.5% in large cities), it appears the financial security of the home is more important to the mental health and well-being of men than women. Furthermore, women in our sample had an average annual income of only 1300 USD (7920.14 Yuan, SD = 14,962.23) in 2010, while men reported a mean annual income of about 2000 USD (12,708.08 Yuan SD = 14,977.20).

**Table 6:** Regression of Mental Health Score on Enabling Factors

	Female	Male
Constant	17.96	17.44
Close relationship with neighbor	−.510*	−.409*
Current paid employment	.658*	.001
Own a home	−.053	−.693*
Child providing social or financial support	.126*	−.028
Qualify for Old age resources	−1.198*	−1.635*
Have medical insurance	−1.053*	.632
Oldest old Senior benefits	.517	.919*
Sample N	1471	1621

Note: *Indicates to significant factors, *p* < .05 or < .01 level

Emotional and financial support from children is common in China with 99% of the sample having at least 1 child and 93.3% reporting at least some form of support from children. The value of this support in predicting mental health and well-being seems to vary dramatically as women reported worse mental health and well-being when such help was received, perhaps suggesting that women receiving such care may in fact be in worse condition than men, for whom there was no effect. While men may be able to rely on their spouse, older women without a spouse turn to their children as the social convoy model might predict.

For both men and women pensions greatly improved mental health and well-being, as this may enable more financial flexibility to meet old age expenses. Medical insurance has a similar positive effect for women, as it may enhance access to medical services and reduce any associated financial burden. Interestingly, no significant effect is seen for men, who generally do not utilize medical services as often.

Finally, receiving the special pension payment available only to those 80 and above is associated with worse mental and well-being among men, but has no significant effect for women. This may be because the oldest men consider themselves to be in worse general condition relative to their expectations and have more life challenges at ages 80 and above than their female counterparts. Table 5 presents the enabling factors on different gender respectively.

#### Need factors

The fourth hypothesis proposed having daily care needs or needing to delay medical treatment would be strong predictors of mental health scores and was largely supported except for the objective measure of number of chronic diseases. Need factors are described in Table 7. Not surprisingly, nearly all of the factors associated with poor health are related to mental health and well-being, except for the number of chronic diseases. Even though the effects of having daily care needs and positive self-rated health are strong and statistically significant related with bad mental health for both men and women, however women have higher effects on these categories. On the contrary, delayed medical treatment will influence more negatively on their mental health and well-being for men than women. The effects of having daily care needs and positive self-rated health or needing to delay medical treatment are so strong and negate the effect of actual chronic disease diagnosis. While one might expect disease to be associated with mental health and well-being, long lasting diseases themselves will not necessarily lead to stress or anxiety that might reduce mental health and well-being (Table 7).

**Table 7:** Regression of Mental Health Score on Need Factors

	Female	Male
Constant	11.62	11.73
Daily care needs	.612^a^	.510^a^
Self-rated health	.939^a^	.761^a^
Delayed medical treatment in last 3 years	.832^a^	1.30^a^
Number of chronic diseases	.048	.040
N	1459	1614

Note: ^a^ Indicates to significant factors, *p* <.05 or <.01 level

## Discussion

This study explored how an approach designed to measure health services utilization might also work in understanding the lives of those facing in the challenges of older age. Exploring the impact of environmental and social characteristics on perceptions of mental health and well-being can be understood from a framework that addresses predisposing, enabling, and need factors.

**Predisposing factors** involve mainly issues outside of one’s direct control that define the opportunities available to either enhance or decrease quality of life. Our findings demonstrated that many predisposing factors greatly influenced mental health measures, with cumulative effects of early life opportunities (e.g., education level or being from a middle province), as well as recent events (e.g., widowhood, and ability to manage income) impacting self-perception. These characteristics cannot be affected by policy or interventions, yet play an important role in self-perception day to day and have been shown to influence use of mental health services after natural disasters [31] as well as predict depression in Hong Kong [32]. Region of residence in China may impact mental health of older adults because their family members often migrate to higher paying work in neighboring cities and provinces [33] leaving older adults who remain with the double-edged sword of poverty and a lack of nearby family support.

**Enabling factors** differed greatly between men and women as reflected in their unique life course experiences. The cumulative impact of these lifestyle differences between men and women is most clearly seen at older ages as the widening gaps in health and mental health appear between those who have lived under better versus worse circumstances. There may be an interesting period effect, where this generation, exposed to dramatic changes in standards of living in China, demonstrates unique resilience despite limited resources in current old age. Our results concur with other studies which find those with strong social connections generally have better mental health outcomes [34, 35]. In some cases, a resource such as qualifying for policies available to older adults actually has a negative association with mental health, suggesting some policies in China may be targeted only to those who need it most, but are not generous enough to positively impact reports of well being. Furthermore, while men may be able to rely on their spouse, older women without a spouse turn to their children as the social convoy model [36] might predict.

**Need factors** affect reported mental health scores of men and women similarly with more physical limitations having a negative affect of mental health as has been found by others exploring the linkages between mental and physical health in the United States [37, 38]. Delayed medical care seeking is a common issue in China, particularly for those with Alzheimer’s or similar issues [39]. Being unable to access mental health treatment can create a cycle where the condition declines further. Need factors illustrate core issues which identify risk for depression or other serious condition, and yet are often unmet among older adults in China.

## Conclusion

Mental health and sense of well-being is an issue with direct impact on individuals, families, and the communities in which they live. China illustrates a unique model when exploring resilience and the impact of older age challenges on mental health. Older people in China today have lived through dramatic social and environmental change as China developed from a largely poor farming society into a highly diversified mix of modern and rural life styles.

Nearly all older women across China have some dependency on family support due to rising costs of living and very limited savings or current income to support their old age. Declines in family size and the diaspora of family members seeking employment strains that dependence on family. The modernization of China has left older adults with financial and social stress. The usual coping mechanisms depend on family resources for social and financial support. Filial piety and intergenerational solidarity on which many older adults depend on are thought to be on the decline [40]. In many families paid caregivers substitute for the caregiving role once expected to be fulfilled by eldest son’s or their wives. For the well-educated or higher income, paid caregivers can serve the daily physical needs, and often unintentionally serve the emotional needs as well. For those with less income, and yet who must live apart for income, support is dependent on neighbors and more distant relatives, the quality of that support likely varies widely.

The current study also provided evidence that older adults’ mental health and well-being may differ by gender. Since some factors have a positive influence on men but the opposite effect on women, the issue of gender differences should be considered in understanding social support and social events [5] and in understanding the impact of pensions [41].

Finally, there are several limitations of the study. The cross-sectional data can only test associations, and the survey asked a limited number of relevant questions regarding lifestyle and mental health. The Andersen model can most easily be applied to smaller scale community data, where need and enabling factors can be closely linked back to specific communities. The data used are nationally representative of a large country which has enormous diversity of life experiences and various local cultural issues which are difficult to control for adequately. Nonetheless, the ecological factors at the macro level remain important, and more locally focused studies can explore the nuances which predict resilience and sense of well-being in older populations adapting to rapid societal change.

## Data Availability

The data that support the findings of this study are available from the China Women’s Federation but restrictions apply to the availability of these data, which were used under license for the current study, and so are not publicly available. Data are however available from the authors upon reasonable request and with permission of the China Women’s Federation.

## Abbreviations

NGO: Non-government organization
IBM SPSS: A statistical analysis package for the social sciences owned by IBM corporation
